# Intestinal Inflammation Induced by Soybean Meal Ingestion Increases Intestinal Permeability and Neutrophil Turnover Independently of Microbiota in Zebrafish

**DOI:** 10.3389/fimmu.2020.01330

**Published:** 2020-07-24

**Authors:** Camila J. Solis, M. Kristina Hamilton, Mario Caruffo, Juan P. Garcia-Lopez, Paola Navarrete, Karen Guillemin, Carmen G. Feijoo

**Affiliations:** ^1^Fish Immunology Laboratory, Faculty of Life Sciences, Universidad Andres Bello, Santiago, Chile; ^2^Millennium Nucleus in the Biology of Intestinal Microbiota, Santiago, Chile; ^3^Institute of Molecular Biology, University of Oregon, Eugene, OR, United States; ^4^Escuela de Biotecnología, Facultad de Ciencias, Universidad Santo Tomás, Santiago, Chile; ^5^Laboratory of Microbiology and Probiotics, Institute of Nutrition and Food Technology (INTA), University of Chile, Santiago, Chile; ^6^Humans and the Microbiome Program, Canadian Institute for Advanced Research, Toronto, ON, Canada

**Keywords:** neutrophil turnover, innate immunity, epithelium permeability, germ free, tight junctions

## Abstract

Intestinal inflammation is a condition shared by several intestinal chronic diseases, such as Crohn's disease and ulcerative colitis, with severely detrimental consequences in the long run. Current mammalian models have considerably increased understanding of this pathological condition, highlighting the fact that, in most of the cases, it is a highly complex and multifactorial problem and difficult to deal with. Thus, there is an increasingly evident need for alternative animal models that could offer complementary approaches that have not been exploited in rodents, thereby contributing to a different view on the disease. Here, we report the effects of a soybean meal–induced intestinal inflammation model on intestinal integrity and function as well as on neutrophil recruitment and microbiota composition in zebrafish. We find that the induced intestinal inflammation process is accompanied by an increase in epithelial permeability in addition to changes in the mRNA levels of different tight junction proteins. Conversely, there was no evidence of damage of epithelial cells nor an increase in their proliferation. Of note, our results show that this intestinal inflammatory model is induced independently of the presence of microbiota. On the other hand, this inflammatory process affects intestinal physiology by decreasing protein absorption, increasing neutrophil replacement, and altering microbiota composition with a decrease in the diversity of cultivable bacteria.

## Introduction

Inflammatory bowel diseases (IBD), such as Crohn's disease (CD) and ulcerative colitis (UC), have emerged as a major health problem worldwide (1). A highly prevalent characteristic in IBD patients is the malfunction or loss of the integrity of the intestinal barrier, which can be observed as an increase in permeability and epithelial damage (2) and, in some cases, accompanied by decreased expression or redistribution of tight junction proteins, including occludins, claudins, and junctional adhesion molecules (3). A central factor associated with IBD is the intestinal microbiota since many studies have revealed that patients with IBD have a microbiota composition different from that of healthy individuals, characterized by a reduction in butyrate-producing obligate anaerobes from the Firmicutes phylum and a large increase in facultative anaerobes from the Proteobacteria phylum (4). However, it remains unclear if microbial dysbiosis contributes to or is a consequence of intestinal inflammation. One key aspect of IBD is that the intestinal mucosa is damaged by an increase in number and activity of neutrophils (5). Neutrophils not only secrete a variety of tissue-damaging factors, including reactive oxygen species, pro-inflammatory lipid mediators, and proteases, but may also negatively influence tissue repair (6). Furthermore, it has been shown that neutrophils in patients with IBD display delayed apoptosis (7), thus promoting tissue damage. Indeed, in UC, the extent of neutrophil infiltration correlates with the severity of the disease and is included in the UC severity scoring system (8). In contrast, some reports attribute beneficial properties to neutrophils during IBD (9, 10). However, it is not clear if these contradictions are due to different experimental strategies or to different neutrophil subsets involved in IBD development (5).

Importantly, many studies of IBD patients are conducted retrospectively, resulting in the inability to determine if the observed gut alterations were a causal event in the etiopathogenesis of IBD or merely the consequence of profound changes in physiology due to the ongoing inflammatory response. In this sense, rodent IBD models have considerably contributed to the understanding of the disease, but the exact contributions of the different risk factors are still unknown as well as the molecular mechanisms responsible for the observed physiological alterations. In this context, complementary approaches to those used until now, difficult to carry out in rodents, such as live imaging and high-throughput screening, can be performed using alternative animal models.

We proposed the use of zebrafish (*Danio rerio*) as an alternative model to study intestinal inflammation to complement the already available rodent models. It is important to highlight that zebrafish have a conserved intestinal architecture and physiology with mammals in addition to all classic immune cell types (11). Moreover, in some respects, zebrafish show comparative advantages with respect to rodents, such as optical transparency and an increasing number of transgenic lines with labeling of specific cell types. The combination of these two features allow the study of intestinal inflammation process *in vivo*, in real time, in the whole animal without any surgery and with labeled cell types of interest. Previously, we developed a strategy to induce intestinal inflammation based on the intake of a diet composed mainly of soybean meal and quantification of the inflammation process by neutrophil presence (12). Characterization of the leukocyte response indicates that the inflammatory process triggered in this way is T cell–dependent, and the transcription level of key cytokines suggest a Th_17_ profile (13). In the present study, we evaluated the effect of the soybean meal–based diet on other critical aspects, such as the integrity of the intestinal epithelium barrier, tight junction protein expression, protein absorption, intestinal neutrophil turnover, and intestinal bacterial microbiota composition. Our findings show that the intestinal inflammation triggered in this manner is characterized by an increase in epithelial permeability and changes in mRNA levels of tight junction proteins; however, epithelial cell damage was observed. Importantly, this intestinal inflammation is induced independently of the presence of microbiota. Finally, as a consequence of the inflammatory process, we observed a decrease in protein absorption capacity, an increase in neutrophil turnover, and a modification of the bacterial microbiota composition with a decrease in the diversity of cultivable bacteria.

## Results

### Intestinal Inflammation Induced by Soybean Meal Increases Intestinal Permeability, Alters Tight Junction Protein mRNA Levels, and Decreases Protein Absorption

**Figure 1:**
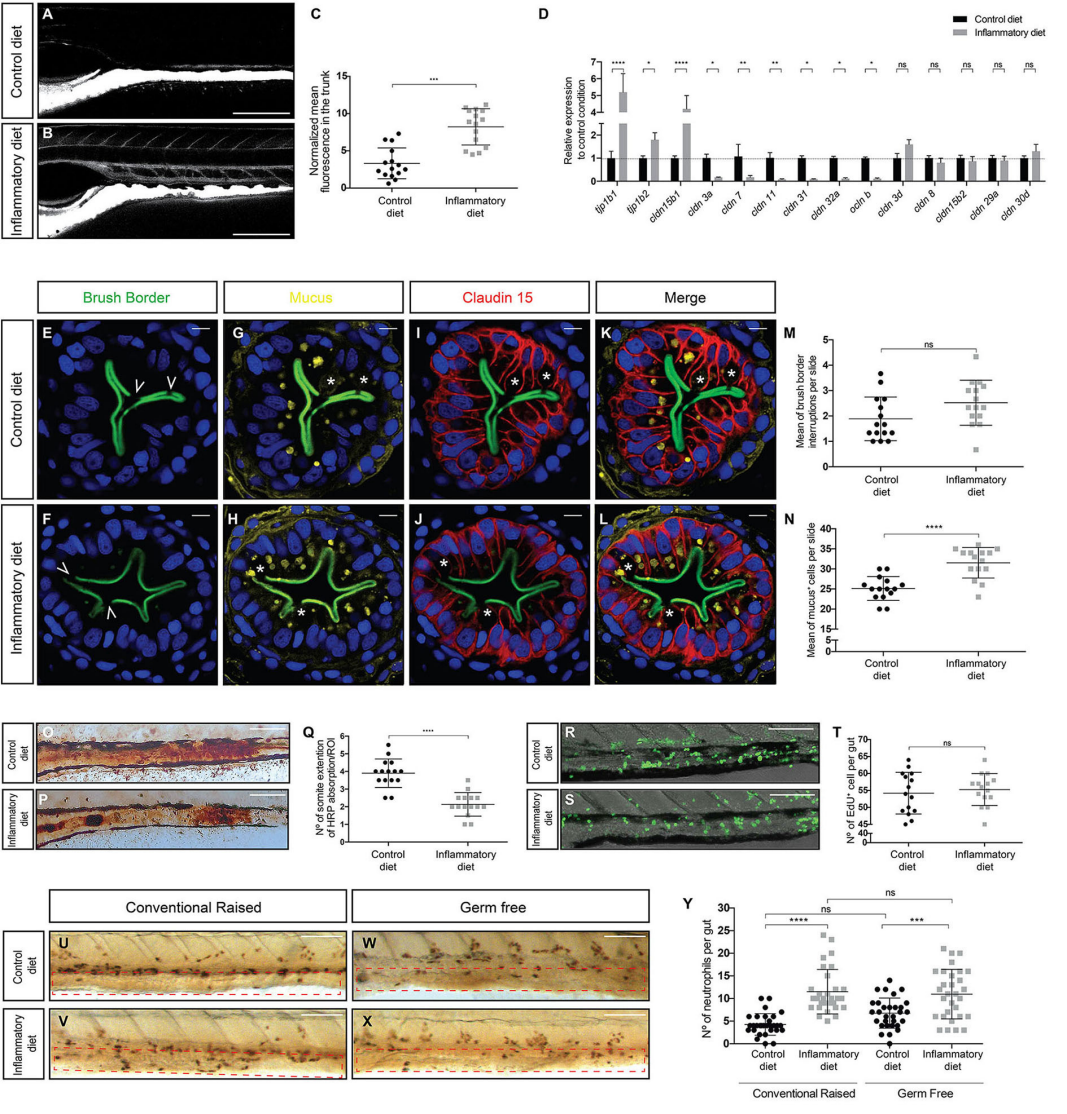
Intestinal inflammation induced by soybean meal alters intestinal physiology and is independent of the presence of microbiota. **(A,B)** Lateral view of the mid-intestine of 9 dpf larvae showing the diffusion of dextran in control **(A)** and inflamed **(B)** larvae. Scale bar, 200 um. **(C)** Normalized dextran fluorescence quantification in the trunk of control and inflamed larvae. **(D)** Relative mRNA expression of several tight junction proteins. All data was normalized against *rpl13a* and compared to the control condition (dotted line). **(E–L)** Transversal cryosection of the midintestine of 9 dpf control and inflamed larvae. **(E–J)** Immunofluorescence labeling the brush border **(E,F)**, mucus **(G,H)**, and Claudin 15 **(I,J)**; nuclei were stained with DAPI (blue). **(K,L)** Merge of the four channels in control and inflamed larvae. Scale bar, 5 um. **(M,N)** Quantification of brush border interruptions (white arrowheads in **E,F**) and goblet cells (white asterisks in G and H). **(O,P)** Representative images of the area of the midintestine showing HRP absorption in control and inflamed larvae. Scale bar, 100 um. **(Q)** Quantification of the area covered by HRP in the midintestine of control and inflamed larvae. **(R,S)** Representative images showing the number of cell that proliferate during 16 h in control and inflamed larvae. Scale bar, 100 um. **(T)** Quantification of EdU^+^ cells in the midintestine per larva. **(U–X)** Lateral view of the midintestine showing immunohistochemistry against the neutrophil marker Mpx on those conventionally raised. The area quantified is delimited by the red dotted rectangle **(U,V)** and germ-free larvae **(W,X)**. Scale bar, 100 um. **(Y)** Quantification of the amount of neutrophils present in the midintestine in conventionally raised and germ-free larvae. Permeability, immunofluorescence, protein absorption, proliferation assay, and immunohistochemistry were performed at least in three biological replicates with 15 larvae of 9 dpf per condition. Statistical analysis was performed using the Mann–Whitney *U*-test, ***p < 0.001, ****p > 0.0001. RT-qPCR was performed at least in three biological replicates with 100 intestines from 9 dpf larvae per condition. Statistical analysis was analyzed using one-way ANOVA and Tukey multiple comparison test. ns, non-significant, *p < 0.05, **p < 0.01, and ****p < 0.0001.

In a prior work, we demonstrated how ingestion of a soybean meal–based diet induces a T cell–dependent intestinal inflammation process in zebrafish larvae (12, 13). An important and uncharacterized aspect in this intestinal inflammation model is the integrity of the epithelial barrier. We took advantage of the TgBAC(cdn15la-GFP)pd1034 transgenic line, which expresses a fluorescently labeled Claudin 15 tight junction protein, labeling the contour of enterocytes (14) to detect any alterations. To determine changes in permeability, we performed an *in vivo* assay, where fluorescently labeled dextran was directly introduced into the gut by microgavage, and 30 min later, the dye distribution was determined. In control larvae, dextran remained in the gut lumen (Figure 1A), whereas in inflamed larvae, dextran breached the intestinal epithelium and diffused into the circulatory system (Figure 1B). Quantification of the normalized mean fluorescence in the trunk, an area where no fluorescence should be observed, indicated that the inflamed gut had increased permeability (Figure 1C). Next, we explored the possibility that the observed increase in permeability was related to changes in the level of proteins that are members of the tight junction complex; thus, we evaluated the mRNA levels of *claudin (cldn) 3a, 3d, 7, 8, 11, 15b1, 15b2, 29, 30d, 31, and 32a*; *occludin (ocln) B* and tight junction proteins (*tjp) 1b1* and *1b2* in the gut. Our results indicated that mRNAs encoding Tjp1b1, Tjp1b2, and Cldn15b1 were significantly increased in inflamed larvae compared to controls (Figure 1D). In contrast, transcripts encoding Cldn 3a, 7, 11, 31, 32a, and Ocln b significantly decreased their level in inflamed larvae. We detected no significant changes in mRNA levels of Cldn 3d, 8, 15b2, 29a, or 30d (Figure 1D). We then analyzed if the ingestion of soybean meal would induce epithelial damage. We prepared transverse histological sections from the midintestine and evaluated the brush border integrity by immunofluorescence. Our results show that both control and inflamed guts had the same amount of brush border interruptions with an average of two per slide (Figures 1E,F,M), interruptions that coincide with the presence of a goblet cell that had released its mucus content into the intestinal lumen (Figures 1E–H,K,L). Due to data reporting an increase in the amount of mucus during intestinal inflammation (15), we evaluated the number of mucus^+^ cells (i.e., goblet cells) in control and inflamed larvae and found that the latter displayed significantly more goblet cells, 32 cells per slide per larva, in contrast to control larvae, which showed 25 per slide per larva (Figures 1G,H,N). Finally, we compared the expression pattern of GFP, which represents Claudin 15, between control and inflamed larvae, and no differences were observed (Figures 1I,J). In summary, these results show that ingestion of soybean meal increases intestinal permeability, alters mRNA levels of several tight junction proteins, and increases the number of goblet cells but does not lead to epithelial histopathology.

To analyze if soybean meal–induced inflammation alters intestinal function, we examined one of the key functions of the intestinal epithelium: protein absorption. We incubated larvae with horse peroxidase (HRP) for 2 h as the sole protein source and then fixed to histochemically detect HPR within the apical region of specialized enterocytes in the midintestine (16). In the control situation, robust HRP staining was detected in a section of the gut, covering an average of 4 somites (Figures 1O,Q). Likewise, in the inflamed gut, HRP stain was also present but in a narrow region, comprising an average of 2 somites (Figures 1P,Q). We also evaluated if the inflammation process affected the proliferation rate of intestinal epithelial cells and, with this, epithelial replacement. To this end, we incubated larvae with the thymidine nucleoside EdU for 16 h and then detected its incorporation into newly synthesized nuclear DNA. The results showed that the number of proliferating cells detected in the guts of control larvae was indistinguishable from that observed in the guts of inflamed larvae (Figures 1R–T). Thus, our results suggest that the inflamed guts decrease their protein absorption capacity but retain normal proliferation rates, indicating that the epithelium is not undergoing inflammation-induced hypertrophy.

### Intestinal Inflammation Induced by Soybean Meal Occurs Under Germ-Free Conditions

At present, there are intestinal inflammatory models where the microbiota is a key player in the induction of inflammation as is the case of the DSS model (17, 18). Thus, to determine the function of the intestinal microbiota in the induction of the inflammatory process, we performed the feeding protocol under germ-free conditions (16). The results show that the number of neutrophils present in the intestine increased in conventionally bred larvae, corroborating what was previously described (12) in both control and inflamed larvae with an average of 4 neutrophils in the control condition and 10 in the inflamed condition (Figures 1U,V,Y). Similarly, in a germ-free environment, the number of neutrophils present in the intestines of control larvae reached an average of 6 neutrophils and in inflamed larvae 11 neutrophils (Figures 1W–Y), indicating that an inflammatory process was underway. Thus, these results reveal that our inflammatory model is independent on the presence of intestinal microbiota because, in germ-free larvae, inflammation is still triggered.

### Intestinal Inflammation Induced by Soybean Meal Increases Neutrophil Turnover

**Figure 2:**
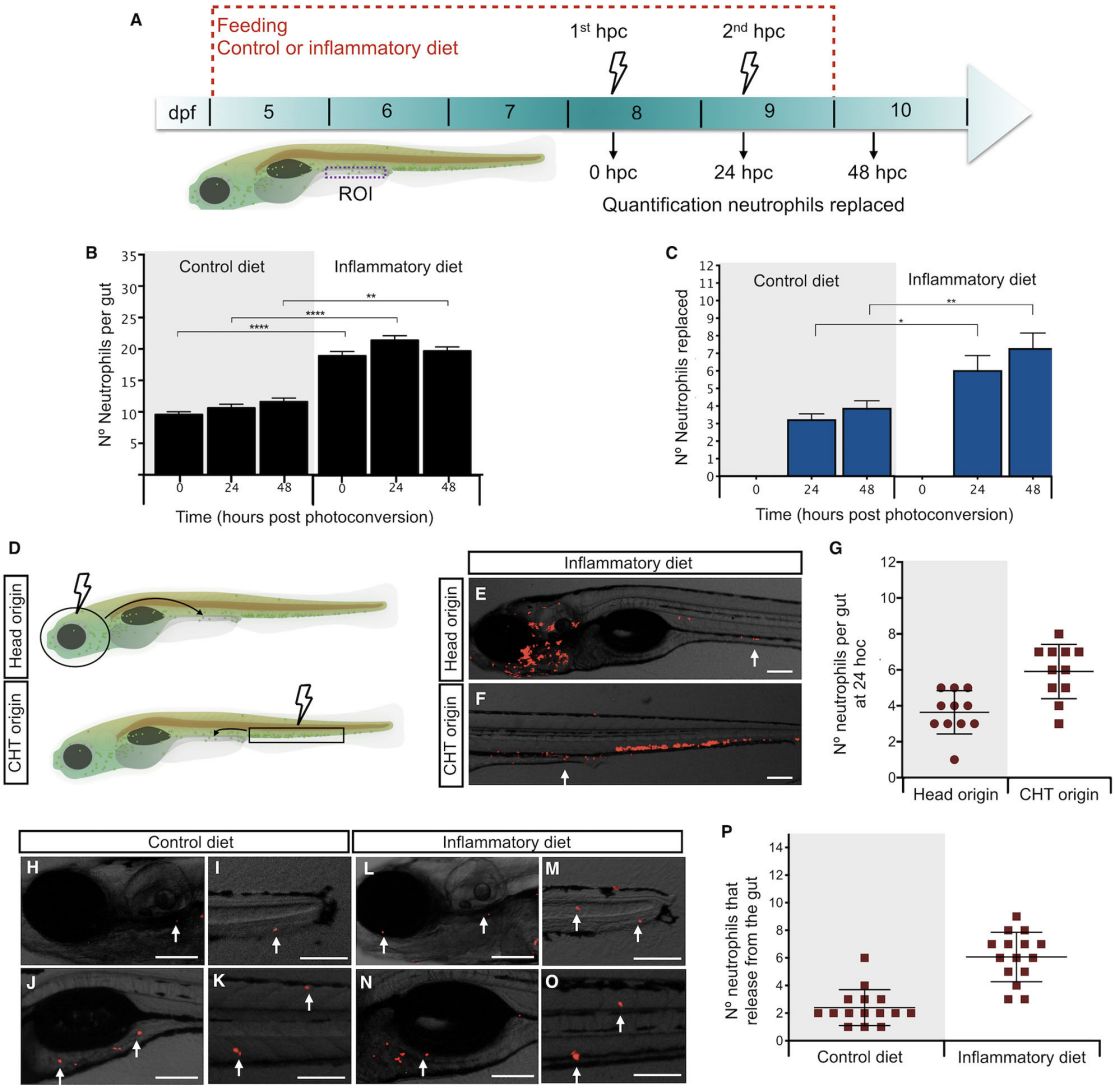
Intestinal inflammation induced by soybean meal increases neutrophil turnover. **(A)** Experimental strategy used in **(B,C,H–P)**. Larvae at 5 dpf were fed with control or inflammatory diet for 6 days. Then, 1st and 2nd photoconversion of neutrophils present in the intestine was performed at 8 and 9 days post-fertilization (dpf), respectively. Quantification of red- and green-neutrophils present at midintestine (purple dotted line) at 0, 24, and 48 h post-photoconversion (hpc) was performed. **(B)** Quantification of the total amount of neutrophils present in the intestine at 0, 24, and 48 h post-photoconversion (hpc) in control and inflamed larvae. **(C)** Quantification of the number of neutrophils replaced at 24 and 48 hpc in control and inflamed larvae. **(D)** Experimental strategy used in **(E–G)**. The head (circle) or caudal hematopoietic tissue (CHT, rectangle) region was photoconverted at 8 dpf larvae. Later, at 24 hpc red-head-neutrophils and red-CHT-neutrophils present in the intestine were quantified. **(E,F)** Representative images showing red-head-neutrophil or red-CHT-neutrophils infiltrated in the intestine at 24 hpc in inflamed larvae. **(G)** Quantification of head and CHT photoconverted neutrophils in the intestine after 24 hpc. **(H–O)** Representative images showing the region of the body (head, caudal fin, anterior intestine, trunk) where intestine-derived neutrophils were found. **(P)** Quantification of the number of neutrophils that left the intestine at 24 h. Statistical analysis was performed with the Mann–Whitney *U* test. ns., non-significant, *p < 0.05, **p < 0.01, and ****p < 0.001. Scale bar, 200 um.

In a previous report, we demonstrated that the soybean meal–based diet induced a persistent neutrophil infiltration into the midintestine of zebrafish larvae (12). To understand the dynamics of neutrophil recruitment to the gut, we took advantage of the Tg(mpxI:Dendra2)^uwm4Tg^ transgenic line, in which the fluorescence of transgenic neutrophils can be switched from green to red after photoconversion of the Dendra2 protein (19). Thus, we induced the inflammatory process and performed two photoconversions, every 24 h, quantifying neutrophils before and after each photoconversion (Figure 2A). Green neutrophils in the gut represent those newly arrived during a period of 24 h, and red neutrophils outside the gut are those that left the intestine during a period of 24 h (Supplementary Figures 1A–L). At 0 h post-photoconversion (hpc), we observed an average of 10 neutrophils in control larvae and 19 in inflamed larvae (Figure 2B). At 24 hpc, we found that, in control larvae, the number of total neutrophils remained similar with 11 present in the gut (Figure 2B). Importantly, an average of 3 neutrophils were replaced (Figure 2C), representing 30% of total neutrophils. Likewise, in the case of inflamed larvae, 21 neutrophils were present in the gut, and 6 neutrophils were replaced (Figures 2B,C), corresponding to 29% of total neutrophils in the gut. A similar situation in control and inflamed larvae was observed at 48 hpc where 4 and 7 neutrophils were replaced, respectively (Figure 2C). Although the percentage of replacement was similar in both conditions, the total amount of substituted neutrophils in the inflamed condition was at least double compared to those changed in the control condition at 24 and 48 hpc (Figure 2C). These results show that neutrophil presence in the intestine is dynamic and that neutrophils are replaced continually and in greater numbers in the inflamed condition. On the other hand, it appears that, during inflammation, the observed neutrophil increase was due to a rise in cells recruited to the intestine rather than caused by the proliferation of neutrophils that were already present there. To test this hypothesis, we photoconverted the two regions were neutrophils are mainly located in zebrafish larvae: the head (where more differentiated neutrophils are present) and the caudal hematopoietic tissue (CHT, were non-activated or more undifferentiated neutrophils are located). Then, after 24 hpc, we quantified red neutrophils present in the gut (Figure 2D). We found that, in the inflammatory condition, an average of 4 head-red neutrophils and 6 CHT-red neutrophils were in the gut (Figures 2E–G). In the case of the control condition, as expected, the replaced neutrophils were recruited from the head and CHT (Supplementary Figures 1M–P). This result confirms that, during inflammation, the new neutrophils in the intestine are recruited from other tissues.

Next, we hypothesized that neutrophils that leave the intestine during inflammation have a different destination compared to controls; thus, we decided to determine their location at 24 hpc. After applying the strategy described in Figure 2A, we observed that, in the control and inflammatory conditions, neutrophils that were previously present in the gut were now located at the head, tail, somites, caudal hematopoietic tissue (CHT), and anterior intestine (Figures 2H–P), suggesting that there is no preferred region for each specific group.

### Intestinal Inflammation Induced by Soybean Meal Results in Modification of Dominant and Cultivable Bacterial Microbiota

**Figure 3:**
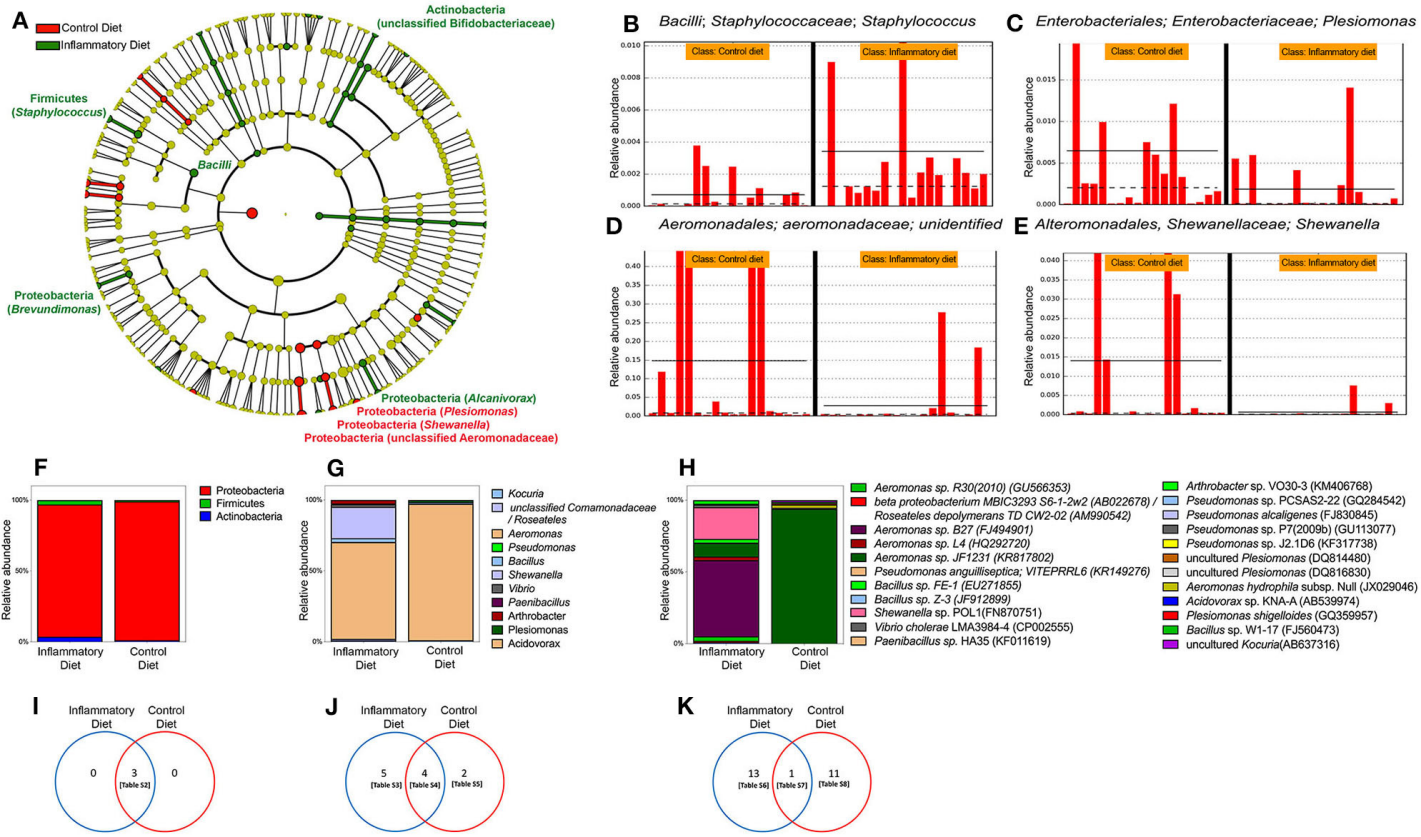
Gut microbiota changes induced by soybean meal diet. **(A)** Cladogram showing significant changes in genus relative abundance in 9 dpf zebrafish larvae labeled [phylum, (*genera*)] unless otherwise noted. Circle color indicates change in abundance: yellow, no change; red, elevated in fishmeal (FM); and green, elevated in soybean meal (SBM). Linear discriminant analysis (LDA) effect size analysis (LEfSe) score >3 was considered significant. QIIME data analysis was used. **(B–E)** The four most significant taxa. The horizontal solid straight line in each panel indicates the group means, and the dotted line indicates the group medians. **(F–H)** Analysis of cultivable microbiota, relative abundance of phyla. **(I–K)** Venn diagrams showing exclusive or shared phyla, genera and species respectively. Tables S2–S8 are detailed in Supplementary Material.

To explore the microbiomes of inflamed and control larvae, independent of culturing, we performed 16S rRNA amplicon sequencing on individual larval intestines. When we compared the resulting bacterial profiles, we found that overall control and inflamed fish did not have significantly different distributions of the Shannon index, a measure of phylogenetic diversity (α-diversity), or non-metric multidimension scaling (NMDS) using the Jaccard similarity coefficient (β-diversity). Of the 186 genera detected in larvae gut, 18 changed significantly in relative abundance, depending on the diet, fishmeal or soybean meal (Figure 3A). All three of our analytic techniques showed that inflamed larvae had a significantly increased relative abundance of the Bacilli family compared to control larvae. Specifically, in the Bacilli family, QIIME analysis showed that Staphylococcus was significantly increased (Figure 3B). Interestingly, inflamed larvae also had increased relative abundance of an unclassified genus in the *Bifidobacteriaceae* family. All other significant differences were found in the *Proteobacteria* phylum, showing that inflamed larvae had increased relative abundance of *Brevundimonas* and *Alcanivorax* and a decreased relative abundance of *Plesiomonas, Shewanella*, and an unclassified genus in the *Aeromonadaceae* family (Figures 3A,C–E). Next, we determined if the soybean meal alters the composition of intestinal cultivable bacterial microbiota. For this purpose, we cultured control and inflamed guts and sequenced the 16S rRNA genes of cultured bacteria. Similar bacterial counts were detected in control and inflamed intestine, 10^4.4^ and 10^4.5^ CFU/larvae, respectively (*p* < 0.05, Mann–Whitney test). We recovered *Proteobacteria, Actinobacteria*, and *Firmicutes* with similar relative abundance in inflamed and control larvae (*p* < 0.05), with a marked dominance of the *Proteobacteria* phylum in both groups (Figures 3F,I). More differences were observed at the genus and species level although no significant differences were observed in relative abundances between both groups (Figures 3G,H,J,K). Only one abundant *Aeromonas* was shared between groups. In the control group, we identified different species of *Plesiomonas* and *Pseudomonas* (Supplementary Table 1). *Acidovorax* sp., uncultured *Kocuria*, and *Aeromonas hydrophila* were also detected. As we observed in the direct microbiota analysis, *Aeromonas* and *Plesiomonas* were most prevalent in control larvae. In inflamed larvae, we identified *Kocuria* sp., uncultured *Roseateles, Bacillus* sp., *Vibrio cholerae, Paenibacillus, Arthrobacter* sp., and different *Aeromonas* and *Shewanella* species. These results showed that inflamed larvae compared to control larvae harbored different bacterial species (Supplementary Table 1).

## Discussion

In the present work, we characterize the effects induced by the ingestion of a soybean meal diet on intestinal epithelial barrier integrity and microbiota composition as well as the role of gut microbiota in inducing the inflammation process. We found that soybean meal increases intestinal permeability, presumably by altering the composition of the tight junction protein complex but without leading to overt pathology of the brush border or to epithelial hyperplasia. Moreover, we found that the extent of inflammation generated in this manner is sufficient to impair intestinal protein absorption capacity and increase neutrophil turnover in the intestine. Importantly, the inflammation shown is independent of the presence of commensal bacteria even though we show soybean meal–induced intestinal bacterial population changes. These results suggest that, in our intestinal inflammation model, inflammation is a primary response to the presence of soybean meal and a driver of bacterial microbiota composition modification although we cannot exclude a possible effect of the diet in the microbiota composition.

Increased intestinal permeability is a main feature of many diseases, including IBD (20) as well as several mouse model of enteritis (21–23). In our case, we found increased permeability together with changes in transcript abundance of several tight junction proteins. Numerous reports have demonstrated the effect of tight junction alterations in intestinal permeability. In a mouse model of T cell–induced acute diarrhea, intestinal permeability was associated with the rearrangement of cellular distribution of occludin and, to some extent, proteins of the junctional adhesion molecules family (24). Likewise, it is reported that patients with active Crohn's disease or ulcerative colitis and impaired intestinal permeability have decreased expression of occludin as well as claudin 4, 5, 7, and 8, whereas claudin 2 is strongly upregulated (25). In agreement with this evidence, we observed that occludin b and claudin 7 were downregulated as were other claudins, such as claudin 3a, 11, 31, and 32a. In addition, a previous report in another fish species, turbot, indicated that the ingestion of a soybean meal–based diet increased intestinal permeability (26). By analyzing histological sections of the intestine, the authors found that intercellular junctions were disrupted in soybean meal–fed fish soybean meal, resulting in an increased intercellular space. Likewise, in another previous study where weaned piglets were fed a soybean-extracted lectin (agglutinin), Zhao et al. found that the expression of intestinal epithelial ZO-1 declined, leading to increased intestinal permeability (27). Thus, considering these reports and the fact that soy protein does not induce intestinal inflammation or epithelial barrier dysfunction in zebrafish, but saponin, a glucoside present in soybean, does (12), we hypothesize that the mechanism by which a soybean meal diet induces intestinal inflammation is through compounds, such as lectins and saponins, that impair the expression of several proteins that form part of the tight junction complex, thus increasing intestinal permeability.

One important aspect of our intestinal inflammation model is that it is induced independently of the presence of microbiota. In general, in mouse models of colitis where the epithelial barrier is severely disrupted due to chemical compounds or genetic defects, such as in the DSS-colitis model (28, 29), N-cadherin dominant negative mice (30), Muc1/2^−/−^ mice (31, 32), Mdr1a^−/−^ mice (33), or in those animals where the immune response is compromised due to the genetic background, such as in IL10^−/−^ and Stat3^−/−^ mice (34, 35), inflammation is absent when animals are maintained under germ-free conditions. Of note, when animals are removed from their gnotobiotic condition, mice rapidly develop an inflammatory process (36). In these cases, the commensal microbiota induce the inflammation process, mainly due to the gross abnormalities of the gut epithelial barrier that allow bacteria to be present in the lamina propria or due to a defective mucosal T cell response (21). This requirement for microbiota to drive the inflammatory process is also observed in a zebrafish genetic model of spontaneous intestinal inflammation (22). In contrast, we observed an increased neutrophil number in the intestine of animals with normal genetic background when maintained under germ-free conditions, indicating that soybean meal is directly responsible for triggering the inflammatory process. The observed differences in microbiota between the soybean meal–fed and control larvae were subtle and likely arose as a consequence of the differences in the intestinal environment between these two groups with no significant differences in phylogenetic diversity. Using three independent analytic approaches, we showed that bacteria in the Bacilli class were increased in soybean meal–fed larvae, in particular Staphylococcus. Inflammation causes physiological changes in the intestine, such as availability of oxygen and other electron acceptors, that can drive overgrowth of certain pathogenic bacterial groups, including Proteobacteria, and create positive feedback loops of dysbiosis (37). Interestingly, the Proteobacteria genera *Shewanella* was decreased in soybean meal–fed larvae. Previous work in zebrafish has shown that *Shewanella* may play a protective role against inflammation (38). In our analysis, significant shifts in particular bacterial genera, rather than the overall community composition, were observed, and whether these shifts are reproducible in response to the mentioned intestinal physiological parameters or others remains to be determined.

On the other hand and in agreement with this study, previous works have shown that the inclusion of soybean meal in the fish diet did not alter intestinal bacterial counts (39). Of note, the shift in observed cultivable species has been correlated with the inclusion of soybean meal and not with the intestinal inflammation condition (39). In addition, a dominant sequence identified as *Bifidobacterium* has been detected in the pelleted feed of Atlantic salmon containing 10% soybean meal (40). In our study, similar sequences (uncultured *Bifidobacteriaceae*) were more abundant in soybean meal–fed larvae, suggesting that this vegetable component could select this microorganism in feed and/or in our soybean meal–fed larvae. Further analyses must be performed in order to elucidate whether these bacterial shifts are related to soybean meal inclusion or intestinal inflammation.

The soybean meal diet induced physiological changes in the intestine that could be observed as a decrease in protein absorption and increase in neutrophil turnover and dysbiosis. Malabsorption of macronutrients, such as fat, and micronutrients, such as Vitamin B12, Vitamin D (17), and trace elements (18), has been reported in patients with Crohn's disease. The most likely reason for this is the loss of intestinal absorptive surface and malfunction of the mucosa. In the case of neutrophil infiltration in the intestine, we observed that not only the number of intestinal neutrophils is increased, but also their daily replacement. In agreement with this result is the fact that the extent of neutrophil infiltration correlates with the severity of ulcerative colitis (8), and patients with active ulcerative colitis display an increased neutrophil-to-lymphocyte ratio in the blood (41). Neutrophil contribution to the pathogenesis of IBD remains controversial; some studies describe a beneficial role, and others report detrimental effects (10, 42, 43). One explanation for this fact could be that different neutrophils subsets are involved in active IBD (9). Notably, massive neutrophil transmigration across the intestinal epithelial barrier has been shown to alter levels of tight junction proteins, thereby increasing epithelial permeability and facilitating the arrival of more neutrophils to the gut, thus triggering an uncontrolled positive feedback amplification loop leading to tissue damage and resolution delay (45).

Further investigation is needed to clearly understand the specific weight of each risk factor in the etiopathogenesis of IBD and the physiological effects that they induced in the gut. Thus, based on the conserved intestinal and immune response between mammals and zebrafish, our intestinal inflammatory model is a suitable tool to contribute to deciphering the mechanisms underlying this complex disease and to provide a rational basis for the development of more effective and targeted therapeutic interventions.

## Materials and Methods

### Ethics Statement

All animal handling procedures were approved by the “Committee of Animal Bioethics of the Universidad Andres Bello,” Certificate number 007/2016.

### Zebrafish Husbandry

Adult zebrafish were maintained at the fish facility of the Universidad Andres Bello, following standard protocols (46). TgBAC(cdn15la-GFP)pd1034 (14) and Tg(mpxI:Dendra2)^uwm4Tg^ (19) embryos were obtained by natural spawning and maintained at 28.5°C in E3 medium (5 mM NaCl, 0.17 mM KCl, 0.33 mM CaCl_2_, 0.33 mM MgSO_4_, pH 7.0).

### Feeding Strategy

Experimental feeding was conducted as was previously described by Hedrera et al. (12) with some modification. Briefly, 30 larvae were maintained in 100 ml of aquarium water and fed with a fishmeal-based diet (from now on control diet) or a soybean meal–based diet (from now on inflammatory diet) (Supplementary Table 9) twice a day from 5 to 8 days post-fertilization (dpf). After the feeding strategy, larvae were changed to fresh aquarium water and left for 19 h without food to promote intestinal emptying.

### Photoconversion and *in vivo* Neutrophil Quantification

Fifteen Tg(mpxI:Dendra2)^uwm4Tg^ (19) larvae were fed from 5 to 9 days post-fertilization (dpf) according to the protocol described above. At 8 and 9 dpf, larvae were anesthetized with 0.017% tricaine and embedded in 2% methylene cellulose to performed neutrophil photoconversion. The midintestine was incised with ultraviolet light (350–400 nm) at 90% potency for 1 min of exposure under a fluorescence microscope (Olympus BX51) using minimum aperture diaphragm. Then the number of green and red neutrophils present in the midintestine were quantified right after photoconversion and at 8, 9, and 10 dpf. Neutrophil replacement was determined according to the number of red neutrophils found outside the intestine and green neutrophils present the intestine (Supplementary Figure 1).

### Histological Sections and Immunolabeling

Nine dpf larvae were anesthetized and fixed in PFA (4% para-formaldehyde in phosphate buffered saline) overnight. Larvae were embedded in 1.5% low melting agarose, dehydrated in 30% sucrose solution overnight at 4°C and cut in into 30 μm slices using a Leica CM1510S cryostat. Cryosectioned tissue was refixed with PFA for 20 min, followed by successive washes in 1% DMSO. Tissue was incubated in blocking solution (1% BSA, 1% DMSO, 5% Goat Serum) for 1 h and incubated overnight with either anti-brush border (Abcam) or anti GFP (ThermoFisher Scientific). Secondary antibody Alexa 488, Alexa 594, or Alexa 647 (Molecular Probes) was used. Mucus staining was performed with WGA (Molecular Probes). Sections were mounted in SlowFade (Life Technologies) and analyzed under an Olympus FluoView FV1000 Spectral Confocal Microscope. The mean of brush border interruptions and mucus positive cells per slide in each larva was plotted. Fifteen larvae were analyzed per condition in three independent experiments.

### Permeability Assay

Oral microgavage was performed as previously described (47) with minor modifications. Briefly, 15 larvae exposed to the experimental feeding strategy were anesthetized, mounted in 2.5% methylcellulose (Sigma) and gavaged with ~5 nL of 1% solution of FITC-dextran (4,000 MW; Sigma). After 15 min, larvae were imaged under an Olympus FluoView FV1000 Spectral Confocal Microscope. Images were processed using ImageJ software to quantify the fluorescence density in the intestine and in the somites dorsal to this region. As a control, a background region was measured outside of the fish and subtracted from the density quantified in the somite region. All values were normalized to the average value from each control group.

### Quantitative Polymerase Chain Reaction (qPCR)

One hundred intestines per diet were processed for total RNA extraction using NucleoSpin® RNA Xs (Macherey-Nagel) according to the manufacturer's instructions, and RNA integrity was corroborated by denaturing agarose electrophoresis. cDNA was synthesized from 2.5 μg of total RNA using SuperScript II Reverse Transcriptase and Oligo-dT primers (ThermoFisher Scientific). Primer sequences are indicated in Supplementary Table 10. qPCR was performed as was previously described (12). The mean Ct values from each sample were normalized against the mean Ct value of a reference gene (*rpl13a*). All experiments were done in triplicate and with three biological replicates.

### Proliferation Assay

Larvae were exposed to the nucleotide analog 5-ethynyl-2′-deoxyuridine (EdU) as previously described (48) with some modifications. Larvae were immersed in 100 μg/ml EdU (Invitrogen) with 0.5% DMSO for 16 h and fixed overnight at 4°C in 4% PFA. Larvae were then processed using the Click-iT EdU Imaging Kit (Invitrogen). EdU-labeled nuclei within the intestine were quantified in at least 30 larvae per condition in three independent experiments.

### Protein Absorption Assay

Protein absorption was measured as previously described (49). Briefly, after 19 h of starvation following the experimental feeding strategy, larvae were incubated overnight in 2 mg/ml HRP (Sigma) and fixed in PFA. HRP activity was detected using Vector HRP substrate kit (Vector Labs) and the area covered by the mark was measured as number of somites.

### Gnotobiotic Generation and Feeding Strategy Under Germ-Free Conditions

Germ-free larvae were generated as previously described (16, 50). At 5 dpf, germ-free larvae began to be fed with UV-sterilized diets, following the same strategy of conventional feeding. Five larvae were sampled each day, homogenized and incubated in Tryptic Soy Broth without antibiotics for 16 h to check bacterial growth.

### Neutrophil Immunodetection

Immunohistochemistry was performed as previously described (13), using a myeloperoxidase (Mpx) primary antibody (Genetex) followed by its detection with anti-rabbit peroxidase (Sigma). The number of neutrophils infiltrated in the midintestine was quantified as was previously described (12, 13). A minimum of 30 larvae were analyzed per condition in three independent experiments.

### Identification of Cultivable Aerobic Gut Microbiota

Ten intestines per treatment in duplicate were extracted and resuspended in 250 μl PBS. 10-fold serial dilutions were plated onto trypticase soy agar and incubated for 3 days at 28°C in duplicate. All colonies were isolated, purified, and identified by sequencing the 16S rRNA gene, as described below. DNA from each morphologically different colony was extracted. Amplification of the gene coding 16S rRNA gene performed with the primers 27F 5′- AGAGTTTGATCCTGGCTCAG-3′ and 1492R 5′-ATTTGCTAAAGCGGGAATCT-3′ as previously described (51). PCR reactions were performed in a reaction mixture containing 1X GoTaq®Green Master Mix (Promega, Madison, WI, USA) and 0.25 pmol/μl of each primer. PCR products were purified and sequenced by Macrogen USA sequencing service. Sequences were edited and cleaned using BIOEDIT software (http://www.mbio.ncsu.edu/BioEdit/bioedit.html). Each sequence was analyzed to find GenBank sequences with the closest BLAST-N hits.

### Microbial DNA Extraction and Sequencing

Intestines of 9 dpf larvae fed either fishmeal or soybean meal were dissected and flash-frozen in liquid nitrogen. Samples were shipped to University of Oregon to be analyzed. DNA was extracted using the PowerMag Microbiome RNA/DNA isolation kit (MoBio, Cat 27500-4-EP), using the Geno/Grinder 2010 for homogenization and epMotion 5075 TMX (eppendorf) liquid handler, according to the manufacturer's protocol. Amplification was performed on the V4 region of the 16S rRNA genes via PCR using the following primers: F515 (5′-GTGCCAGCMGCCGCGGTAA-3′) and R806 (5′-GGACTACHVGGGTWTCTAAT-3′) (52). High-throughput sequencing was performed on 37 DNA samples with Illumina HighSeq paired-end 150-bp runs at the University of Oregon Genomics, Cytometry and Imaging Core.

### Microbial Bioinformatic Analysis

Quantitative Insights Into Microbial Ecology (QIIME, version 1.9.1) (53) software was used for demultiplexing and quality filtering of raw sequences. Operational taxonomic unit (OTU) picking was performed using an open reference workflow script with Greengenes 97 (version 13_8) for taxonomy assignment at 97% similarity. Chimera sequences were identified via Chimera Slyer (54) and removed. With the use of additional filtering, low-read OTUs were also removed (55). Of the 84,505,228 reads analyzed, 84,461,129 reads were used after filtering, resulting in 343 observed OTUs. Diversity, weighted UniFrac principal coordinate analysis (PCoA), and taxa summaries were created through QIIME (core_diversity_analyses.py) with sequencing depth and maximum rarefaction depth set at 300,000. Relative abundance outputs were between 3.52 × 10^−7^ and 97.27%. Alpha and beta diversity were analyzed using QIIME (compare_alpha_diversity.py & make_distance_boxplots.py). Multivariable statistical analysis was performed in the LEfSe package (56) with default parameters. To validate QIIME analysis approach and results, raw sequences were analyzed using DADA2 (57) and phyloseq as we described (44).

### Statistical Analysis

Dextran diffusion, immunofluorescence, protein absorption, proliferation, and neutrophil quantification were analyzed using Mann–Whitney *U*-test. mRNA expression was analyzed using one-way ANOVA and Tukey multiple comparison test. All analyses were performed using Prim 6 (GraphPad Software). The significance level was set to *P* < 0.05 or below.

## Data Availability Statement

All datasets generated for this study are included in the article/Supplementary Material.

## Ethics Statement

The animal study was reviewed and approved by Ethics Committee from the Universidad Andres Bello, Certificate number 007/2016.

## Author Contributions

CS, CF, MC, PN, MH, and KG contributed to the conception and design of the study. CS, JG-L, MC, and MH developed the experiments and performed statistical analysis. CF wrote the first draft of the manuscript. All authors contributed to revising the manuscript, reading, and approving the submitted version.

## Conflict of Interest

The authors declare that the research was conducted in the absence of any commercial or financial relationships that could be construed as a potential conflict of interest.
