# Supplementary material for: Intestinal Inflammation Induced by Soybean Meal Ingestion Increases Intestinal Permeability and Neutrophil Turnover Independently of Microbiota in Zebrafish
Source: Front Immunol. 2020 Jul 24;11:1330. doi: 10.3389/fimmu.2020.01330 (PMC7393261; doi:10.3389/fimmu.2020.01330)
Supplement: Supplementary file 1 [file Data_Sheet_1.docx]

Supplementary Material


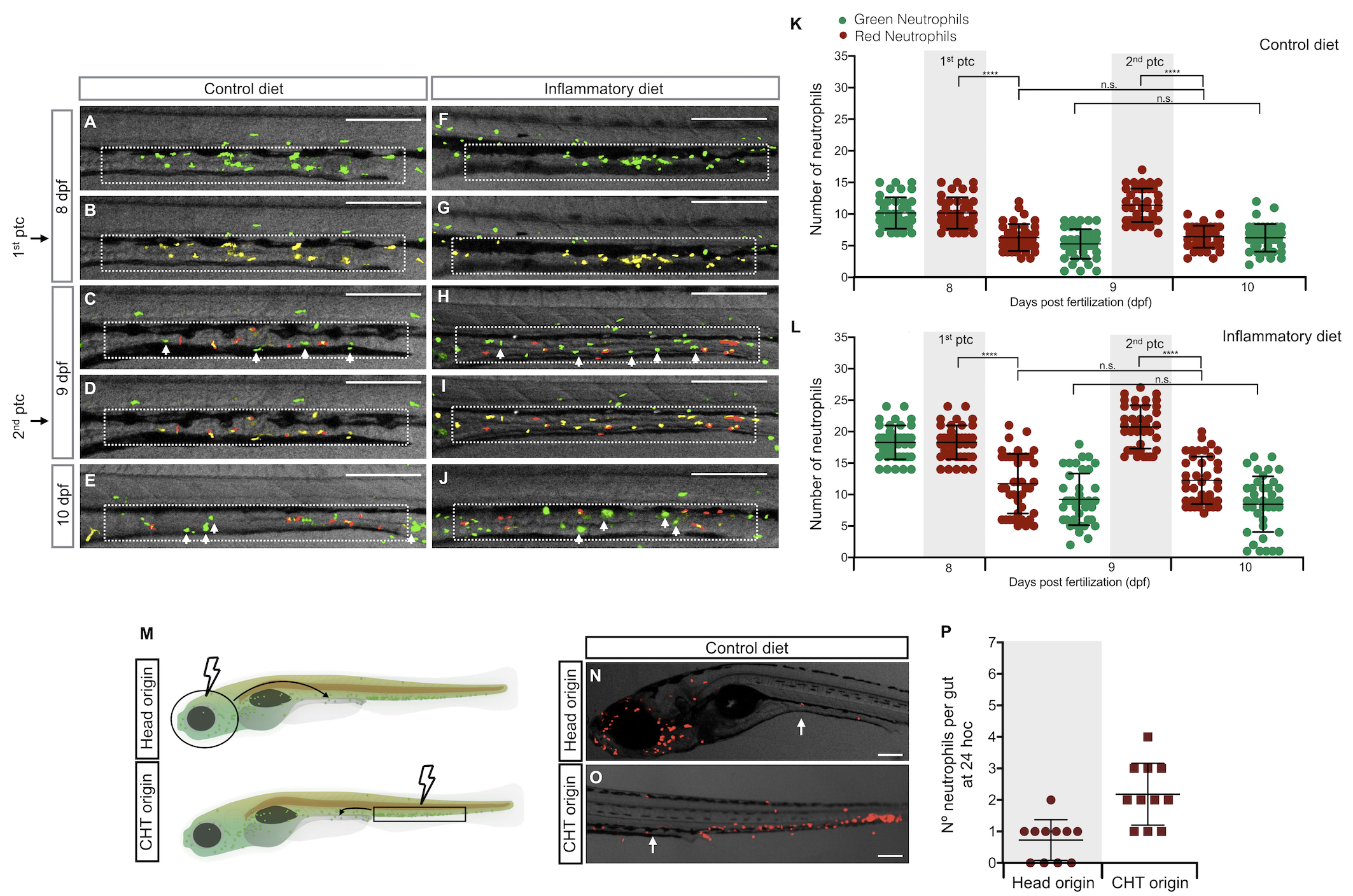


**Supplementary Figure 1. Daily neutrophil recruitment to control and inflamed intestines.** (A-J) Representative images showing green and red neutrophils recruited in control and inflamed larvae. (K, L) Quantification of red and green neutrophils per intestine in control and inflamed larvae respectively. (M) Experimental strategy used in N-P. The head (circle) or caudal hematopoietic tissue (CHT, rectangle) region were photoconverted at 8 dpf larvae. Later, at 24 hpc red-head-neutrophils and red-CHT-neutrophils present in the intestine were quantified. (N) Representative images showing red-head-neutrophil or (O) red-CHT-neutrophils infiltrated in the intestine at 24hpc in control larvae. (P) Quantification of head and CHT photoconverted neutrophils in the intestine after 24hpc. Statistical analysis was performed with the Mann-Whitney U test. n.s.: non-significant, ****p<0,001. Scale bar, 200um.

**Supplementary Table 1.** Nearest-match identification of 16S rDNA sequences obtained from bacterial isolates of cultivable gut microbiota, with known sequences in the RDP release 11 database.

| Name | Diet | Accesion  number | % identity | Affiliation phylum/class | Closest sequence |
| --- | --- | --- | --- | --- | --- |
| Colony 01 | Inflammatory | MN371813 | 99.1 | Actinobacteria/Actinobacteria | *Kocuria* sp*.* ljh-23 (GU217697) |
| Colony 07 | Inflammatory | MN371814 | 99.3 | Proteobacteria/Betaproteobacteria | beta proteobacterium MBIC3293; S6-1-2w2 (AB022678)  *Roseateles depolymerans*; TD CW2-02 (AM990542) |
| Colony 08 | Inflammatory | MN371815 | 97.1 | Proteobacteria/Gammaproteobacteria | *Aeromonas* sp*.* R30(2010) (GU566353) |
| Colony 10 | Inflammatory | MN371816 | 98.7 | Proteobacteria/Gammaproteobacteria | *Aeromonas* sp*.* B27 (FJ494901) |
| Colony 12 | Inflammatory | MN371817 | 99.1 | Proteobacteria/Gammaproteobacteria | *Pseudomonas anguilliseptica*; VITEPRRL6 (KR149276) |
| Colony 15 | Inflammatory | MN371818 | 98.4 | Proteobacteria/Gammaproteobacteria | *Aeromonas* sp*.* L4 (HQ292720) |
| Colony 16 | Inflammatory | MN371819 | 99.4 | Firmicutes/Bacilli | *Bacillus* sp*.* FE-1 (EU271855) |
| Colony 17 | Inflammatory | MN371820 | 99.8 | Proteobacteria/Gammaproteobacteria | *Shewanella* sp*.* POL1(FN870751) |
| Colony 18 | Inflammatory | MN371821 | 99.1 | Proteobacteria/Gammaproteobacteria | *Vibrio cholerae* LMA3984-4 (CP002555) |
| Colony 19 | Inflammatory | MN371822 | 99.7 | Proteobacteria/Gammaproteobacteria | *Vibrio cholerae* LMA3984-4 (CP002555) |
| Colony 20 | Inflammatory | MN371823 | 95.7 | Firmicutes/Bacilli | *Bacillus* sp*.* Z-3 (JF912899) |
| Colony 24 | Inflammatory | MN371824 | 99.4 | Firmicutes/Bacilli | *Paenibacillus* sp*.* HA35 (KF011619) |
| Colony 25 | Inflammatory | MN371825 | 99.3 | Proteobacteria/Gammaproteobacteria | *Aeromonas* sp*.* JF1231 (KR817802) |
| Colony 26 | Inflammatory | MN371826 | 99.3 | Proteobacteria/Gammaproteobacteria | *Shewanella* sp*.* POL1(FN870751) |
| Colony 30 | Inflammatory | MN371827 | 99.5 | Proteobacteria/Gammaproteobacteria | *Shewanella* sp*.* POL1(FN870751) |
| Colony 31 | Inflammatory | MN371828 | 99.3 | Actinobacteria/Actinobacteria | *Arthrobacter* sp*.* VO30-3 (KM406768) |
| Colony 34 | Control | MN371829 | 99.8 | Proteobacteria/Gammaproteobacteria | *Pseudomonas* sp*.* PCSAS2-22 (GQ284542) |
| Colony 35 | Control | MN371830 | 99.6 | Proteobacteria/Gammaproteobacteria | uncultured *Plesiomonas* (DQ814480) |
| Colony 36 | Control | MN371831 | 98.4 | Proteobacteria/Gammaproteobacteria | *Aeromonas* sp*.* JF1231 (KR817802) |
| Colony 39 | Control | MN371832 | 99.6 | Proteobacteria/Betaproteobacteria | *Acidovorax* sp*.* KNA-A (AB539974) |
| Colony 40 | Control | MN371833 | 98.7 | Proteobacteria/Gammaproteobacteria | *Pseudomonas alcaligenes* (FJ830845) |
| Colony 41 | Control | MN371834 | 99.1 | Proteobacteria/Gammaproteobacteria | *Plesiomonas shigelloides* (GQ359957) |
| Colony 42 | Control | MN371835 | 98.3 | Proteobacteria/Gammaproteobacteria | *Pseudomonas* sp*.* P7(2009b) (GU113077) |
| Colony 47 | Control | MN371836 | 97.6 | Firmicutes/Bacilli | *Bacillus* sp. W1-17 (FJ560473) |
| Colony 49 | Control | MN371837 | 99.4 | Proteobacteria/Gammaproteobacteria | *Pseudomonas* sp*.* J2.1D6 (KF317738) |
| Colony 51 | Control | MN371838 | 99.1 | Proteobacteria/Gammaproteobacteria | *Plesiomonas shigelloides* (GQ359957) |
| Colony 55 | Control | MN371839 | 97.4 | Proteobacteria/Gammaproteobacteria | uncultured *Plesiomonas* (DQ816830) |
| Colony 58 | Control | MN371840 | 99.4 | Proteobacteria/Gammaproteobacteria | uncultured *Plesiomonas* (DQ814480) |
| Colony 59 | Control | MN371841 | 93.5 | Actinobacteria/Actinobacteria | uncultured *Kocuria*  (AB637316) |
| Colony 62 | Control | MN371842 | 97.6 | Proteobacteria/Gammaproteobacteria | *Aeromonas hydrophila* subsp. Null (JX029046) |

**Supplementary Table 2.** Composition of the bacterial microbiota of inflamed and control intestines through 16S rRNA sequencing at the Phyla level.

|  | **Relative abundance** | |
| --- | --- | --- |
| **Phyla** | **Inflammatory diet** | **Control diet** |
| Actinobacteria | 3.56 % | 1.09 % |
| Proteobacteria | 93.36 % | 97.96 % |
| Firmicutes | 3.07 % | 0.97 % |

**Supplementary Table 3.** Composition of the bacterial microbiota of inflamed intestines through 16S rRNA sequencing at genus level.

| **Diet** | **Species** | **Relative abundance** |
| --- | --- | --- |
| Inflammatory | *unclassified Comamonadaceae / Roseateles* | 1.02 % |
| Inflammatory | *Shewanella* | 22.13 % |
| Inflammatory | *Vibrio* | 1.81 % |
| Inflammatory | *Paenibacillus* | 0.51 % |
| Inflammatory | *Arthrobacter* | 2.54 % |

**Supplementary Table 4.** Composition of the bacterial microbiota of inflamed and control intestines through 16S rRNA sequencing at the genus level.

|  | **Relative abundance** | |
| --- | --- | --- |
| **Species** | **Inflammatory diet** | **Control diet** |
| *Kocuria* | 1.02 % | 1.09 % |
| *Aeromonas* | 68.15 % | 96.16 % |
| *Pseudomonas* | 0.25 % | 0.15 % |
| *Bacillus* | 2.57 % | 0.97 % |

**Supplementary Table 5.** Composition of the bacterial microbiota of control intestines through 16S rRNA sequencing at the genus level.

| **Diet** | **Species** | **Relative abundance** |
| --- | --- | --- |
| Control | *Plesiomonas* | 1.53 % |
| Control | *Acidovorax* | 0.09 % |

**Supplementary Table 6.** Composition of bacterial microbiota of inflamed intestines through 16S rRNA sequencing at the species level.

| **Diet** | **Species** | **Relative abundance** |
| --- | --- | --- |
| Inflammatory | *Kocuria sp. ljh-23 (GU217697)* | 1.02 % |
| Inflammatory | *beta proteobacterium MBIC3293 S6-1-2w2 (AB022678) / Roseateles depolymerans TD CW2-02 (AM990542)* | 1.02 % |
| Inflammatory | *Aeromonas sp. R30(2010) (GU566353)* | 3.05 % |
| Inflammatory | *Aeromonas sp. B27 (FJ494901)* | 53.15 % |
| Inflammatory | *Aeromonas sp. L4 (HQ292720)* | 2.54 % |
| Inflammatory | *Pseudomonas anguilliseptica; VITEPRRL6 (KR149276)* | 0.25 % |
| Inflammatory | *Bacillus sp. FE-1 (EU271855)* | 2.54 % |
| Inflammatory | *Bacillus sp. Z-3 (JF912899)* | 0.03 % |
| Inflammatory | *Shewanella sp. POL1(FN870751)* | 22.13 % |
| Inflammatory | *Vibrio cholerae LMA3984-4 (CP002555)* | 1.81 % |
| Inflammatory | *Paenibacillus sp. HA35 (KF011619)* | 0.51 % |
| Inflammatory | *Arthrobacter sp. VO30-3 (KM406768)* | 2.54 % |

**Supplementary Table 7.** Composition of the bacterial microbiota of inflamed and control intestines through 16S rRNA sequencing at the species level.

|  | **Relative abundance** | |
| --- | --- | --- |
| **Species** | **Inflammatory diet** | **Control diet** |
| *Aeromonas sp. JF1231 (KR817802)* | 9.40 % | 93.97 % |

**Supplementary Table 8.** Composition of the bacterial microbiota of control intestines through 16S rRNA sequencing at the species level.

| **Diet** | **Species** | **Relative abundance** |
| --- | --- | --- |
| Control | *Pseudomonas sp. PCSAS2-22 (GQ284542)* | 0.07 |
| Control | *Pseudomonas alcaligenes (FJ830845)* | 0.03 |
| Control | *Pseudomonas sp. P7(2009b) (GU113077)* | 0.03 |
| Control | *Pseudomonas sp. J2.1D6 (KF317738)* | 0.02 |
| Control | *uncultured Plesiomonas (DQ814480)* | 0.37 |
| Control | *uncultured Plesiomonas (DQ816830)* | 0.22 |
| Control | *Aeromonas hydrophila subsp. Null (JX029046)* | 2.18 |
| Control | *Acidovorax sp. KNA-A (AB539974)* | 0.09 |
| Control | *Plesiomonas shigelloides (GQ359957)* | 0.94 |
| Control | *Bacillus sp. W1-17 (FJ560473)* | 0.97 |
| Control | *uncultured Kocuria (AB637316)* | 1.09 |

**Supplementary Table 9:** Ingredients and nutrient composition of control and experimental diets.

| **Ingredients g Kg*^-1^*** | **Fish meal (100 FM)** | **Soybean meal (50 SBM)** |
| --- | --- | --- |
| Fishmeal ^a^ | 555 | 250 |
| Soybean meal ^a^ | 0 | 500 |
| Wheat grain meal | 255 | 110 |
| Starch ^b^ | 60 | 60 |
| Fish oil ^c^ | 30 | 60 |
| Vitamineral mix ^a,1^ | 20 | 20 |
| Cellulose ^d^ | 80 | 0 |
| Total | 1000 | 1000 |
| **Analytical composition (dry bases, %)** | | |
| Dry matter | 94.42 (±0.13) | 93.10 (±0.17) |
| Crude protein | 43.76 (±0.58) | 45.44 (±2.30) |
| Crude lipids | 6.54 (±0.25) | 7.05 (±0.24) |
| Ash | 9.730 (±0.07) | 8.38 (±0.12) |
| Gross energy (MJ Kg*^-1^*) | 20.0 (±0.06) | 20.2 (±0.06) |

^1^ As recommended by the NRC (1993)

^a^ Provided by Salmofood, Vitapro, Castro, Chile

^b^ Almisa, Caaguazú, Paraguay

^c^ Reuter S.A, Santiago, Chile

^d^ Sigma-Aldrich, St Louis, MO, USA.

**Supplementary Table 10.** Primer sequences for qPCR

| **Gene** | **Accession Number** | **Forward Primer** | **Reverse Primer** |
| --- | --- | --- | --- |
| *rpl13α* | NM_212784 | TCTGGAGGACTGTAAGAGGTATGC | AGACGCACAATCTTGAGAGCAG |
| *claudin 3a* | NM_131767 | GAATGGGCTATTCTGCTCCA | TCACCCTTTTCATCCGTCTT |
| *claudin 3d* | NM_131764 | GTACCCTCCGCAAAGTCGTA | CTTTCAAGGAAAGACTGACAGC |
| *claudin 7* | NM_131637 | CTTGCTCAAAGGGTCAGTCA | GTCCTTTCCAGCTCGTGAAC |
| *claudin 8* | NM_001003733 | CGTTCATACAGCCCTCTCGT | CACACAAACATGCTTGCACA |
| *claudin 11* | NM_131772 | CCACGATGGAGTTACCAGCTA | TGTGTCTGTGTGAGTTTGAGTGTT |
| *claudin 15b1* | NM_001002446 | TTTGTGTGTTATCGTGGCCG | GCCCATCCCAGATACAGACC |
| *claudin 15b2* | NM_001002446 | CAAGACCACTCTGGGTCTGC | CACTTGAGTCCCATCGAGGC |
| *claudin 29a* | NM_180964 | GGGTCGCGCTTATTCTGTTA | TTCCTACACAAACAGGAGACGAT |
| *claudin 30d* | NM_131763 | AGACAGCGGAAAATACACAGC | TGAGCCTCAATGTCCAACAA |
| *claudin 31* | NM_180965 | GGGTCGGTATACACCAGCTT | TCTGCTTTACAAAGACGATCTCA |
| *claudin 32a* | NM_131768 | GAGCCGCCAAATACTACAGC | TTCGCTACCTTAGACGGGTTA |
| *tjp1b1* | XM_021470734 | GCCAGGGAGTAGGAGGAGAA | AACACAAAAATCCGCACGGG |
| *tjp1b2* | XM_021470734 | TGTGAGTGTGGTGATGGGTG | CTCCTCCTACTCCCTGGCAT |
| *occludin b* | NM_001008618 | GACCATTAAGGATGGCCTCA | GCTGAGCAGCACTGACTTTG |
